# Frontal and parietal lobes play crucial roles in understanding the disorder of consciousness: A perspective from electroencephalogram studies

**DOI:** 10.3389/fnins.2022.1024278

**Published:** 2023-01-26

**Authors:** Yesong Liu, Zhaoyi Li, Yang Bai

**Affiliations:** ^1^School of Basic Medical Sciences, Hangzhou Normal University, Hangzhou, China; ^2^Center for Cognition and Brain Disorders, The Affiliated Hospital of Hangzhou Normal University, Hangzhou, China

**Keywords:** frontal, parietal, neural correlates of consciousness, electroencephalogram (EEG), disorder of consciousness (DOC)

## Abstract

**Background:**

Electroencephalogram (EEG) studies have established many characteristics relevant to consciousness levels of patients with disorder of consciousness (DOC). Although the frontal and parietal brain regions were often highlighted in DOC studies, their electro-neurophysiological roles in constructing human consciousness remain unclear because of the fragmented information from literatures and the complexity of EEG characteristics.

**Methods:**

Existing EEG studies of DOC patients were reviewed and summarized. Relevant findings and results about the frontal and parietal regions were filtered, compared, and concluded to clarify their roles in consciousness classification and outcomes. The evidence covers multi-dimensional EEG characteristics including functional connectivity, non-linear dynamics, spectrum power, transcranial magnetic stimulation-electroencephalography (TMS-EEG), and event-related potential.

**Results and conclusion:**

Electroencephalogram characteristics related to frontal and parietal regions consistently showed high relevance with consciousness: enhancement of low-frequency rhythms, suppression of high-frequency rhythms, reduction of dynamic complexity, and breakdown of networks accompanied with decreasing consciousness. Owing to the limitations of EEG, existing studies have not yet clarified which one between the frontal and parietal has priority in consciousness injury or recovery. Source reconstruction with high-density EEG, machine learning with large samples, and TMS-EEG mapping will be important approaches for refining EEG awareness locations.

## 1. Introduction

Researchers have long debated the origin of consciousness and the neural correlates of consciousness. Studies have demonstrated that the global workspace of the sensory areas, namely, the prefrontal and posterior parietal cortices, is highly correlated with the conscious activity of the brain (Giacino et al., 2014). The posterior cortex contains a posterior hot zone for the production of many conscious experiences such as vision, hearing, and touch (Boly et al., 2017; Koch, 2018), which serves as direct evidence that posterior brain regions are associated with human consciousness. Patients who have suffered severe prefrontal damage still retain arousal and awareness, suggesting that the prefrontal cortex should be excluded as a consciousness-dependent cortex (Koch, 2018). However, some researchers believe that damage to most frontal structures unrelated to consciousness does not lead to a loss of consciousness; key structures in the frontal lobe dominate human consciousness (Koenigs et al., 2007; Koch et al., 2016).

Disorder of consciousness (DOC) is an altered state of consciousness caused by damage or dysfunction in parts of the nervous system that regulate arousal and awareness (Schiff and Plum, 2000; Giacino et al., 2014). DOC patients have usually suffered severe brain damage owing to stroke, hypoxia, etc. (Gosseries et al., 2011b,^2014^). Such patients can be in a vegetative state (VS) or a minimally conscious state (MCS). Both states feature high arousal levels; the MCS involves reproducible non-reflexive behavioral responses, whereas the VS [also called unresponsive wakefulness syndrome (UWS)] only involves reflexive behavioral responses to external stimuli. VS/UWS is a clinical syndrome describing patients who fail to show voluntary motor responsiveness under eyes-open wakefulness (Laureys et al., 2010). MCS patients cannot communicate with their environment; however, they show a fluctuating remnant of volitional behavior (Laureys et al., 2004). Furthermore, MCS could be divided into MCS + and MCS-, dependent on their ability to respond to commands, intentionally communicate, and so on (Chennu et al., 2017; Rizkallah et al., 2019). In addition, Thibaut et al. (2021) identified VS/UWS patients with brain activity similar to MCS as MCS*.

The frontal lobe is the control center of speech function and motor behavior; it is further thought to be involved in higher cognition, including memory and executive power (Chayer and Freedman, 2001). The global workspace theory hypothesizes that consciousness emerges by information processing, which propagates input information to the whole brain through two neuronal networks with centers at the frontal and parietal lobes (Koch, 2018). Neuroimaging studies have shown that an improved consciousness level is accompanied by changes in the metabolic rate of the parietal associative cortices (Laureys et al., 1999) as well as increased frontal-related neural connectivity (Jang and Lee, 2015). An electroencephalogram (EEG) is a non-invasive, highly compatible, and portable measure of brain function, and it allows the application of quantitative methods to better understand and interpret consciousness-related patterns (Kondziella et al., 2015). A variety of clinical and basic science studies have found a correlation between the level of consciousness and the EEG characteristics in frontal and parietal brain regions as well as fronto-parietal connections (Bai et al., 2017). However, the methodology and computation of EEG features are complex, and the abstraction of their neurophysiological interpretation limits their traceability to the neural correlates of consciousness. This makes it difficult for clinicians when they translate them into clinical practice.

## 2. Literature search

**FIGURE 1:**
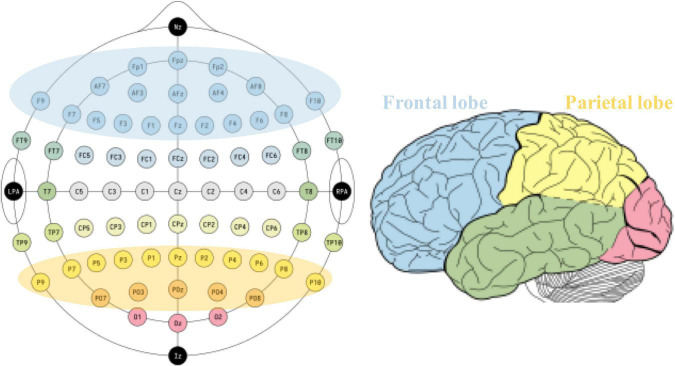
Defined frontal and parietal regions according to typical 10–20 EEG system **(left)** and the corresponding cortex **(right)**.

Literature searches were performed in four electronic databases: EMBASE, MEDLINE (*via* PubMed), Web of Science, and EBSCOAs. The retrieval time limit was set from the establishment of the database to 15 May 2022, and the language was limited to English. The search string was built as follows: EEG OR (Electroencephalogram) AND [(MCS) OR (minimally conscious state) OR (disorder of consciousness) OR (coma) OR (unresponsive wakefulness syndrome) OR (vegetative state) OR (disturbance of consciousness)]. The original search found 1,653 records, with 857 remaining after excluding duplicates. After the preliminary screening of article types, titles, and abstracts, 258 records were left. Ultimately, 43 records were included into our review after excluding literatures that did not explicitly involve specific brain regions. The key findings related to frontal and parietal brain regions were filtered to explore their electrophysiological role in the state of consciousness. Figure 1 shows the defined frontal and parietal regions in EEG 10–20 electrode system and corresponding cortical lobes.

## 3. Evidence of frontal role in DOC

### 3.1. Frontal EEG characteristics in classification of DOC

The frontal lobes of DOC patients showed significantly different spectrum band powers compared with those of healthy controls. They have been proved to be able to distinguish DOC patients and to differentiate them into MCS and VS/UWS patients (Supplementary Table 1). The relative delta and alpha powers in the frontal area showed significant correlations with the revised version of the coma recovery scale (CRS-R) scores (Rossi Sebastiano et al., 2015). DOC patients exhibited a lower frontal delta source pattern compared with that of healthy controls (Naro et al., 2016a). The frontal area of VS/UWS patients showed a higher delta power but lower theta, alpha, and beta (not MCS*) powers than those of MCS* and MCS patients (Rossi Sebastiano et al., 2015; Piarulli et al., 2016; Naro et al., 2018; Thibaut et al., 2021). MCS patients showed lower source magnitudes of beta at frontal lobes than those seen with severe neurocognitive disorders (SNDs) (Leon-Carrion et al., 2008).

The functional network within the frontal area was significantly weaker in DOC patients than in healthy subjects (Naro et al., 2018). The participation coefficient of the frontal cortex positively correlated with the consciousness level (Chennu et al., 2017) and was significantly lower in MCS patients than in healthy controls (Rizkallah et al., 2019). Compared with the control group, the reduced participation coefficient of MCS- and MCS + differed in the frontal lobe and MCS- also showed lower integration in the parietal lobe but not MCS + (Rizkallah et al., 2019). The frontal lobe of DOC patients showed a lower node degree in the theta and alpha bands than in the case of healthy controls (Zhang L. et al., 2022). MCS patients had a higher degree of nodal values (Zhang L. et al., 2022), alpha band quadratic phase self-coupling (QPSC) (Bai et al., 2019), and multiplex clustering coefficients (Cai et al., 2020) at the frontal area than did VS/UWS patients. MCS* also has a higher alpha participation coefficient and alpha degree in the frontal lobe than VS/UWS (Thibaut et al., 2021). Moreover, time-varying gamma phase synchronization was only found in the frontal of MCS patients but not in VS/UWS patients (Naro et al., 2018). Transient states were demonstrated as novel self-constructed brain networks in spontaneous EEG. The anterior state is represented by the high delta power at frontal lobe and enhanced frontal connectivity. The fractional occupancy of the anterior state in DOC patients was significantly higher than that in healthy controls. Specifically, the anterior state occupied the most state expression time in VS/UWS patients and not in MCS patients (Bai et al., 2021).

Vegetative consciousness/unresponsive wakefulness syndrome patients always exhibited lower EEG complexity at the frontal than did MCS and healthy subjects (Thul et al., 2016). It has been revealed through the approximate entropy, amplitude coalition entropy and spectral entropy (Gosseries et al., 2011a; Piarulli et al., 2016; Liu et al., 2021; Visani et al., 2022).

The frontal scalp regions exhibited novel P300 (nP3) in MCS patients but not in VS/UWS patients (Risetti et al., 2013; Naro et al., 2016b). The frontal areas elicited a predictive value mismatch negativity (MMN) which showed a lower average amplitude in DOC patients than in healthy subjects (Hu et al., 2021). In addition, the frontal cluster showed increased delta modulation in command-following patients than in non-command-following patients during the early window of event-related potentials (Rivera-Lillo et al., 2021).

Transcranial magnetic stimulation-electroencephalography (TMS-EEG) could be conducted at the bedside as an advanced stimuli-response technique for improving the diagnostic accuracy of DOC. When TMS is targeted over the frontal region, VS/UWS patients showed simper neural responses and OFF periods, which differed from those of healthy subjects and MCS patients (Rosanova et al., 2012; Ragazzoni et al., 2013). The causal effects of TMS on the local cortical activity were shorter-lived in VS/UWS patients than in healthy awake controls (Rosanova et al., 2018).

### 3.2. Frontal EEG characteristics in outcome of DOC

The frontal functional network could be considered an effective characteristic for tracking consciousness recovery in DOC patients. The frontal QPSC in the theta band predicted patients who recovered their brain functions after 3 months (Bai et al., 2019). Frontal inter-hemisphere coherence in the delta band decreased in patients who recovered after tDCS treatment (Guo et al., 2019). A positive correlation was found between the fronto-central coherence and the motor item improvement of MCS patients (Naro et al., 2016c). Further, the event-related potential was commonly used to track the consciousness recovery of DOC patients (Gosseries et al., 2014). VS/UWS patients with a frontal distribution of nP3 topography recovered to MCS after 4.5 months (Risetti et al., 2013). After 2 weeks of HD tDCS treatment, alpha-beta activity in frontal lobe increased in patients with improved conscious representations (Zhang C. Y. et al., 2022).

## 4. Evidence of parietal role in DOC

### 4.1. Parietal EEG characteristics in classification of DOC

The parietal theta, alpha, and gamma powers were much lower in DOC patients than in healthy subjects (Naro et al., 2016a). The EEG of the parietal region showed a higher delta but lower theta, alpha, and gamma source patterns in VS/UWS patients than those in the healthy subjects (Lechinger et al., 2013; Sitt et al., 2014). Significant correlations exist between the CRS-R scores and the relative delta and relative alpha powers (Rossi Sebastiano et al., 2015). Compared with the parietal lobes of patients with SNDs, those of MCS patients showed a higher amplitude of beta and theta frequencies (Leon-Carrion et al., 2008). Moreover, the parietal gamma oscillatory activity correlates with the level of awareness of DOC patients (Naro et al., 2018). Regarding the difference between VS/UWS and MCS/MCS* patients, the parietal region showed increased delta power but decreased theta, alpha, and beta powers in the former relative to the latter (Naro et al., 2016a,^2018^; Piarulli et al., 2016; Thibaut et al., 2021). The midline of the parietal region had a larger high-to-low frequency power ratio during the day-time than during the night-time in MCS patients, whereas no significant difference was seen in VS/UWS patients (Wislowska et al., 2017). In MCS patients, the parieto-occipital region showed some preserved topographical differentiation of alpha activity, whereas VS/UWS patients showed residual multifocal alpha activities in all regions (Rossi Sebastiano et al., 2015; Naro et al., 2018).

Consistent with the findings in the frontal region, the participation coefficient of the parietal lobe increased greatly with a higher consciousness level (Chennu et al., 2017). The parietal region showed time-varying gamma phase synchronization, higher mutual information, lower multiplex participation coefficient, and higher multiplex clustering coefficients in MCS patients than in VS/UWS patients (King et al., 2013; Naro et al., 2018; Cai et al., 2020). Additionally, MCS* also appeared higher participation coefficient and degree of alpha in the parietal region (Thibaut et al., 2021). Further, the parietal regions of VS/UWS patients showed the most extensive variation of beta bands with higher clustering coefficients compared with those of MCS patients (Cacciola et al., 2019). Furthermore, the fractional occupancy of the posterior transient state, which was characterized by a higher alpha power at parietal lobe and enhanced parietal connectivity, was significantly higher in healthy subjects than in DOC patients (Bai et al., 2021).

When TMS was applied over parietal cortices, VS/UWS patients showed simpler response patterns and OFF periods, unlike MCS patients. The duration of the causal effects of TMS on local cortical activity was shorter-lived in VS/UWS patients than in healthy awake controls (Rosanova et al., 2018). Higher complexity of parietal activities was found in MCS patients than in VS/UWS patients, which were revealed by spectral entropy and permutation entropy (Sitt et al., 2014; Piarulli et al., 2016). VS/UWS patients were found to lack nP3 and P300 components, unlike MCS or conscious patients (Risetti et al., 2013; Xiao et al., 2018).

### 4.2. Parietal EEG characteristics in outcome of DOC

Vegetative consciousness/unresponsive wakefulness syndrome patients exhibited nP3 with parietal scalp topography, and they recovered to MCS after 4.5 months (Risetti et al., 2013). The survivors of DOC showed stronger central-parietal negativity N1 than did the non-survivors (Meiron et al., 2021). The parietal region of patients showed a significant increase in the normalized theta power and an increase in the permutation entropy in the theta-alpha band after tDCS treatment (Hermann et al., 2020). Patients who had prominently parietal strong connections showed negative outcomes (Chennu et al., 2017). After HD-tDCS treatment, alpha-beta increasing and delta decreasing occurred in the parietal lobe (Cai et al., 2019; Zhang C. Y. et al., 2022).

## 5. Frontal-parietal connectivity in DOC

Studies revealed that the functional connectivity between the frontal and parietal brain regions was highly correlated with the state of consciousness of DOC patients (Supplementary Table 1). The fronto-parietal connectivity was impaired in DOC patients (Naro et al., 2020). When referring to behavioral response levels, the strength of the fronto-parietal connectivity in the theta (Lehembre et al., 2012) and alpha (Naro et al., 2018) bands increased with the consciousness level. The inter-hemispheric connectivity in the fronto-parietal cortex of VS/UWS patients was lower than that in MCS patients (Chennu et al., 2017; Cacciola et al., 2019). Although MCS + and MCS- cannot be distinguished from connectivity, MCS + showed a the strongest fronto-parietal focus of topographical pattern for, which is more pronounced in patients with high levels of consciousness (Chennu et al., 2017).

The association of gamma connectivity with consciousness levels was not consistent across studies. An increased fronto-parietal gamma coherence induced by noxious stimulation was reported in healthy subjects and MCS patients but not in VS/UWS patients (Cavinato et al., 2015). However, the gamma coherence decreased after tDCS in MCS patients but not in VS/UWS patients (Bai et al., 2018). Furthermore, a clear TMS-evoked neural response propagated from frontal to parietal in the MCS patients but not in the VS/UWS patients (Wang et al., 2022). In the analysis of transient states, DOC patients showed a break of coherence in the alpha band between the medial prefrontal cortex and posterior cingulate cortex in the anterior state (Bai et al., 2021).

The enhancement of the fronto-parietal connectivity was reported along with the recovery of consciousness. The patients with higher delta, theta, alpha, and beta coherence recovered from VS/UWS to MCS after 1 year (Schorr et al., 2016). Similarly, the fronto-parietal connectivity in the alpha and beta bands appears in patients with an improved state of consciousness after 3 months (Fingelkurts et al., 2013).

## 6. Discussion

The EEG contains consciousness information in different dimensions: rhythmic oscillations (spectrum), functional network, and dynamic non-linearity. In combination with the multi-dimensional information, the EEG features in the frontal and parietal showed a significant difference with different consciousness levels. The spectrum captures the rhythmic spontaneous activity of neuronal populations (Coleman et al., 2005). Impaired consciousness is usually accompanied by spectral abnormalities in the resting state. In either the frontal or parietal brain regions, VS/UWS patients always showed higher delta power and lower theta, alpha, and beta (not MCS*) powers than those of MCS and MCS* patients. Non-linear dynamic theory considers neural networks to be a complex non-linear system. Non-linear dynamics in the time or frequency domain are more straightforward to quantify the complexity of neural activities. Compared with VS/UWS, the non-linearity of MCS was generally more complex in MCS patients in both the frontal and parietal regions. The functional network, which captures the local integration and global synchronization relationships of the cortex, showed more intensity connections, with higher integration in MCS patients than in VS/UWS patients in both the frontal and parietal brain regions. The TMS-evoked neural responses in the frontal and parietal brain regions were highly abnormal in the case of injury of consciousness. VS/UWS patients showed a specific off-period and a shorter causal effect duration than those of healthy subjects (Rosanova et al., 2018).

The frontal and parietal still showed differences in distinguishing consciousness. Regarding the power spectrum, the frontal only showed a difference between MCS and VS/UWS for delta, theta, alpha, and beta. In the parietal area, high-frequency activity (gamma) was also considered a characteristic relevant to consciousness (Naro et al., 2016a,^2018^). The indicator of the parietal gamma activity even exceeded alpha in the deterioration of consciousness (Srivas et al., 2016). Although the functional connectivity showed a decrease in both the frontal and parietal regions of DOC patients, most evidence for the frontal region came from the comparison of DOC and healthy subjects, while the parietal region showed clear differences between VS/UWS and MCS (Sitt et al., 2014; Chennu et al., 2017; Cacciola et al., 2019; Cai et al., 2020).

Overall, the reduction of consciousness is often accompanied by an enhancement of low-frequency rhythms, suppression of high-frequency rhythms, reduction of dynamic complexity, and breakdown of networks in the frontal and parietal brain regions. Although the current studies reported a difference in EEG characteristics between the frontal and parietal brain regions for the classification of consciousness levels, enough evidence is not available to clarify their priority in consciousness injury or recovery.

Literatures reported a significant correlation between the fronto-parietal network and residual consciousness of DOC patients. According to the current knowledge, the consciousness level of DOC patients depends on the strength of the large-scale connectivity between the frontal and parietal brain and is related to the neural activities within local regions. Consciousness impairment is often accompanied by the deterioration and heterogeneity of frontal and parietal connectivity within a certain frequency band (e.g., theta, alpha) (Lehembre et al., 2012; Chennu et al., 2017; Bai et al., 2018; Naro et al., 2018). This is consistent with the meso-circuit model in which the frontal and parietal cortices act as critical hubs and the fronto-parietal connection integrates consciousness-related information processing at the cortical level part of the consciousness circuit (Schiff, 2010; Giacino et al., 2014). The frontal cortex organizes goal-directed behavior (Schiff, 2010), adjusts the body’s arousal levels in different states and alertness, and activates or cooperates with the central thalamus to adapt to higher cognitive needs by increasing activity (Paus et al., 1998; Nagai et al., 2004). The functional connectivity between the frontal and parietal lobes allows the two cortices to not only directly regulate the meso-circuit through feedback but also indirectly through the frontal cortical-striatopallidal-thalamocortical loop systems (Münkle et al., 2000; Werf et al., 2002) to maintain normal conscious pathways in the brain.

In the cross-sectional comparison of different states of consciousness in DOC, most comparisons between VS/UWS and MCS could not clearly exclude impacts from individual differences, such as age, etiology, treatment strategy, and care environment. The longitudinal tracking of DOC outcomes could help to identify the most important characteristics by focusing on the temporal correlation between consciousness levels and neural electrical activity in individuals. The follow-up studies highlighted the role of evoked potentials in the frontal and parietal regions in detecting consciousness. The nP3 topography changed as the patients recovered from VS/UWS to MCS. DOC survivors showed lateralization in the N1 component topography compared to non-survivors. The centers of the topography distribution indicated important information processing in the frontal and parietal regions (Risetti et al., 2013; Meiron et al., 2021). Simultaneous changes of delta, alpha and beta index the patients with signs of consciousness improvement (Cai et al., 2019; Zhang C. Y. et al., 2022). Furthermore, the enhancement of frequency coupling (QPSC) and phase synchronization (coherence) (Naro et al., 2016c; Bai et al., 2019; Guo et al., 2019) in the frontal region both indicated the functional recovery of DOC patients.

Most EEG studies made conclusions based on scalp-level observations, which would be biased by the effect of volume conduction. Although existing studies consistently highlight the role of the frontal and parietal regions in DOC, the accurate location of the NCC at the cortical level remains unclear. Source construction in combination with high-density EEG and individualized anatomy could technically improve the spatial accuracy of EEG characteristics by solving the volume conduction problem. In addition to the source construction, TMS-EEG can provide high cortical-spatial precision causal relationships between TMS targets and neural responses. Current TMS-EEG studies have provided considerable and detailed information for the diagnosis of DOC and cortical damage. However, studies to establish maps between target-evoked responses and consciousness are still needed. These could facilitate our understanding of the excitability and plasticity of the frontal and parietal regions in human consciousness. In EEG studies, source reconstruction of high-density EEG, machine learning with large samples, and TMS-EEG mapping should be important tools for refining EEG awareness locations and locating the smallest neural correlates of consciousness.

## Author contributions

YB wrote the manuscript. YL and ZL reviewed the manuscript. All authors contributed to the article and approved the submitted version.
