# Erratum: Frontal and parietal lobes play crucial roles in understanding the disorder of consciousness: A perspective from electroencephalogram studies

**DOI:** 10.3389/fnins.2023.1167744

**Published:** 2023-03-03

**Authors:** Frontiers Production Office

**Affiliations:** Frontiers Media SA, Lausanne, Switzerland

**Keywords:** frontal, parietal, neural correlates of consciousness, electroencephalogram (EEG), disorder of consciousness (DOC)

Due to a production error, the affiliation of reviewer Yi Yang is incorrect. The affiliation “University Hospital and University of Liège, Belgium” has been changed to: “Beijing Tiantan Hospital Affiliated to Capital Medical University, China.”

The publisher apologizes for this mistake. The original article has been updated.

