# Supplementary material for: Frontal and parietal lobes play crucial roles in understanding the disorder of consciousness: A perspective from electroencephalogram studies
Source: Front Neurosci. 2023 Jan 26;16:1024278. doi: 10.3389/fnins.2022.1024278 (PMC9909102; doi:10.3389/fnins.2022.1024278)
Supplement: Supplementary file 1 [file Table_1.DOCX]

**Table 1.** Frontal and parietal related EEG characteristics in classification and outcomes of disorder of consciousness

| **Frontal** | **Subjects** | **EEG methodology** | **Evidence EEG characteristics** | **Main evidence** |
| --- | --- | --- | --- | --- |
| **Evidence from DOC classification** |  |  |  |  |
| ([Rossi Sebastiano et al., 2015](#_ENREF_28)) | MCS 57  VS/UWS 85 | Resting-state spectrum | Alpha and delta power | 1.The VS/UWS patients had a significantly higher delta relative power in the **fronto-central** than the MCS patients, but a significantly lower alpha relative power.  2.Signifcant correlations between CRS scores and relative delta power and relative alpha power in the **fronto-central** regions. |
| ([Piarulli et al., 2016](#_ENREF_21)) | MCS 6  VS/UWS 6 | Resting-state spectrum  Nonlinear analysis | Delta, theta, alpha and beta power  Spectral entropy | 1.VS/UWS showed higher delta power and lower theta, alpha, beta1 power than MCS at **Fz**.  2.MCS mean spectral entropy was higher than that of the VS/UWS at **Fz**.  3.Spectral entropy time variability is higher in MCS as compared to VS/UWS at **Fz**. |
| ([Naro et al., 2018](#_ENREF_17)) | MCS 15  VS/UWS 17 | Resting-state spectrum  Functional connectivity | Delta and alpha power  dWPLI | 1. VS/UWS show greater **frontal** delta-power than patients with MCS.  2. MCS but not VS/UWS showed time-varying gamma dWPLI at **frontal**. |
| ([Leon-Carrion et al., 2008](#_ENREF_14)) | SND 9  MCS 7 | Resting-state spectrum | Beta power | Lower source magnitudes of beta in **frontal** lobes were found for MCS patients compared to SND. |
| ([Naro et al., 2016a](#_ENREF_18)) | HC 10  MCS 7  VS/UWS 6 | Resting-state spectrum | Delta power | **Frontal** delta source pattern was greater in the HC as compared to MCS and VS/UWS. |
| ([Babiloni et al., 2010](#_ENREF_1)) | HC 13  LIS 13 | Resting-state spectrum | Alpha power | The power of alpha 2 (individual alpha frequency -2 to individual alpha frequency) and alpha 3 (individual alpha frequency to individual alpha frequency+2) sources in **frontal** region was lower in LIS compared to controls. |
| ([Thul et al., 2016](#_ENREF_31)) | HC 24  MCS 7  VS/UWS 8 | Nonlinear analysis  Functional connectivity | Permutation entropy  Symbolic transfer entropy | 1. Permutation entropy between controls and MCS patients showed highest area under receiver operating characteristic curve at **fronto-central** electrodes.  2.MCS showed different symbolic transfer entropy with healthy controls especially in the **fronto-temporal** network. |
| ([Bai et al., 2019](#_ENREF_3)) | MCS 20  VS/UWS 31 | Functional connectivity | QPSC | Patients with MCS had a higher QPSC_alpha value in right **frontal** regions than VS/UWS patients. |
| ([Rizkallah et al., 2019](#_ENREF_25)) | HC 21  EMCS 6  MCS 46  (MCS+ 29  MCS- 17)  VS/UWS 9 | Functional connectivity and network | Participation coefficient | The participation coefficient in the **frontal** cortex was lower in the MCS group than in the control group.  A large number of brain regions had decreased integration in the MCS- group as compared to the control group, including the right **orbitofrontal**, and left superior parietal. The left/right **orbitofrontal**, showed a higher participation coefficient in the theta band for controls than MCS+ patients.  A decrease in participation coefficient in MCS- patients mainly in the right **dorsolateral frontal cortex**, compared with control. A much wider network of regions had a decreased participation coefficient between control and MCS+ groups, mainly located in the left/ right lateral **frontal** cortex. |
| ([Cai et al., 2020](#_ENREF_5)) | HC 23  MCS 19  VS/UWS 35 | Functional connectivity and network | Multiplex clustering coefficient | **Frontal** areas show remarkably increased multiplex clustering coefficient values in MCS and the controls compared to VS/UWS. |
| ([Risetti et al., 2013](#_ENREF_23)) | MCS 3  VS/UWS 8 | Event related potential | Auditory stimulation  nP3 | The nP3 in passive state is predominantly present in the **frontal** scalp regions in MCS, but not in VS/UWS. |
| ([Naro et al., 2016b](#_ENREF_19)) | MCS 7  VS/UWS 7 | Event related potential | Transorbital alternating current stimulation  P300 (visual) | The oddball paradigm induced a P300 (from target stimuli) at **DLPFC** (dorsolateral prefrontal cortex) in the HC, MCS with a visual CRS-R score ≥3, but not in the VS/UWS. |
| ([Visani et al., 2022](#_ENREF_32)) | MCS 13  VS/UWS 19 | Nonlinear analysis | Amplitude coalition entropy  Lempel-Ziv complexity  synchrony coalition entropy | MCS patients compared with VS/UWS patients had significantly higher amplitude coalition entropy values in theta, alpha, and, to a lesser extent, beta bands, prominently at the **fronto-central** region of the left hemisphere. |
| ([Hu et al., 2021](#_ENREF_10)) | HC 20  MCS 12  VS/UWS 2 | Event related potential | MMN (auditory) | MMN was found to be elicited in the **frontal** and central areas in DOC, with lower average amplitude compared with HC. |
| ([Zhang et al., 2022](#_ENREF_36)) | HC 19  MCS 29  VS/UWS 18 | Functional connectivity and network | Coherence | 1. In the theta and alpha bands, the control group had a higher node degree value in the central area of the **frontal** lobe than DOC.  2. The MCS group had a higher degree of nodal value in the right area of the **frontal** lobe, but the VS/UWS group did not show this phenomenon. |
| ([Rivera-Lillo et al., 2021](#_ENREF_24)) | HC 10  MCS 9  VS/UWS 11 | Event related potential | Delta (auditory) | Delta modulation during the early window at the **frontal** cluster showed an increased modulation in command following patients compared with non-command following patients. |
| ([Liu et al., 2021](#_ENREF_15)) | MCS 64  VS/UWS 98 | Nonlinear analysis | Approximate entropy | Under pain stimulation conditions, the values of approximate entropy in **frontal** site were significantly higher in the MCS group than in the VS/UWS group for unaffected hemisphere. |
| ([Gosseries et al., 2011](#_ENREF_7)) | HC 16  MCS 26  VS/UWS 24  Coma 6 | Nonlinear analysis | State entropy and response entropy | MCS patients showed higher EEG entropy values at left **fronto-temporal** than the VS/UWS patients. |
| ([Ragazzoni et al., 2013](#_ENREF_22)) | HC 5  MCS 5  VS/UWS 8 | TMS-EEG | TMS evoked potentials | Compare to VS/UWS and MCS groups, healthy controls has a stronger activation over **frontal** electrodes. |
| ([Rosanova et al., 2018](#_ENREF_26)) | HC 20  VS/UWS 16 | TMS-EEG | TMS evoked potentials | 1. TMS applied over **frontal** cortices, VS/UWS patients OFF-periods were ubiquitously observed and differed from awake healthy subjects stimulated over the same areas.  2. TMS applied over **frontal** cortices, the duration of the causal effects of TMS on local cortical activity in VS/UWS patients was shorter-lived than healthy awake controls. |
| ([Rosanova et al., 2012](#_ENREF_27)) | LIS 2  MCS 5  VS/UWS 5 | TMS-EEG | TMS evoked potentials | TMS at **frontal** lobe evoked a slow, positive-negative EEG response in all VS/UWS patients, instead, MCS patients showed fluctuating signs of non-reflexive reactions to external stimuli, which was comparable to LIS patients. |
| ([Chennu et al., 2017](#_ENREF_6)) | HC 26  LIS 4  EMCS 11  MCS66  VS/UWS 23 | Functional connectivity and network | Participation coefficients | High participation coefficients in **frontal** areas along with increasing level of consciousness. |
| ([Bai et al., 2021](#_ENREF_2)) | HC 25  MCS 25  VS/UWS 37 | Transient state | Fractional occupancy | 1. Compared with the healthy subjects, DOC spent more time in the **anterior** state (activation at frontal lobe).  2. Fractional occupancy of the **anterior** state was greater in VS/UWS than MCS. |
| ([Thibaut et al., 2021](#_ENREF_30)) | HC 33  VS/UWS 11  MCS* 15  MCS 54 | Resting-state spectrum  Functional connectivity and network | Theta，beta and alpha power  participation coefficient | MCS* patients had higher power in theta and alpha bands, lower power in delta band, higher alpha participation coefficient and alpha degree in **frontal** regions, compared to VS/UWS patients. |
| **Anterior** | **Subjects** | **EEG methodology** | **Evidence EEG characteristics** | **Main evidence** |
| **Evidence from DOC outcomes** |  |  |  |  |
| ([Bai et al., 2019](#_ENREF_3)) | MCS 20  VS/UWS 31 | Functional connectivity | QPSC | The **frontal** QPSC_theta value showed significant differences between recovered (CRS-R score increased at least 3 points after 3 months) and unrecovered patients. |
| ([Risetti et al., 2013](#_ENREF_23)) | MCS 3  VS/UWS 8 | Event related potential | nP3 (auditory) | The nP3 evoked during the second recording session displayed a change in the topography, being largely distributed over the **frontal** scalp regions in 2/4 VS/UWS recovery to MCS(recovery time of half and 4 months). |
| ([Meiron et al., 2021](#_ENREF_16)) | HC 10  MCS 9  VS/UWS 1 | Event related potential | N1 (auditory) | N1 component topography of DOC non-survivors was non-central, lateralized to **prefrontal** areas after one year. |
| ([Naro et al., 2016c](#_ENREF_20)) | HC 10  MCS 10  VS/UWS 10 | Functional connectivity | Coherence | A positive correlation between motor item improvement and **fronto-central** theta-coherence for MCS. |
| ([Guo et al., 2019](#_ENREF_8)) | MCS 6  VS/UWS 5 | Functional connectivity | Coherence | Delta coherence decreased in the **frontal** inter-hemisphere regions in DOC patients who recovery after 14 days' high-definition tDCS treatment. |
| ([Wang et al., 2020](#_ENREF_33)) | MCS 9  VS/UWS 2 | Event related potential | MMN | MMN with a **fronto-central** focus, increased with consciousness improvement.. |
| ([Zhang et al., 2022a](#_ENREF_38)) | MCS 29  VS/UWS 13 | Resting-state spectrum | alpha-beta power | Alpha-beta power increased, mainly at the **frontal** electrodes in the recovery patients. |
| ([Guo et al., 2022](#_ENREF_10)) | MCS 20  VS/UWS 13 | Microstate | Occurrence | Occurrence of microstate C (middle **frontal-occipital**) decreased and microstate D (middle **frontal**) increased along with CRS-R improvement. |
| **Parietal** | **Subjects** | **EEG methodology** | **Main EEG characteristics** | **Main evidence** |
| **Evidence from DOC classification** |  |  |  |  |
| ([Rossi Sebastiano et al., 2015](#_ENREF_28)) | MCS 57  VS/UWS 85 | Resting-state spectrum | Delta and alpha power | 1.The VS/UWS patients had a significantly higher delta relative power in the **parieto-occipital** than the MCS patients, but a significantly lower alpha relative power.  2.Signifcant correlations between CRS scores and relative delta power and relative alpha power in the **parieto-occipital**.  3.Alpha activity in MCS patients preserved some topographical differentiation and was mainly represented in the **parieto-occipital** region, whereas the residual alpha activity in the VS patients was uniformly distributed in all regions. |
| ([Lechinger et al., 2013](#_ENREF_12)) | HC 14  MCS 9  VS 8 | Resting-state spectrum | Delta, theta, power | VS/UWS patients had higher delta and theta amplitudes at **Pz** compared to HC |
| .([Sitt JD, 2014](#_ENREF_29)) | HC 14  CS 24  MCS 68  VS/UWS 75 | Resting-state spectrum  Nonlinear analysis | Delta and alpha power  permutation entropy | 1.Normalized theta and normalized alpha power was significantly higher in CS than VS/UWS in **parietal** regions.  2.Complexity was higher in patients with a higher clinical state of consciousness, particularly for a set of electrodes over the **parietal** region.  3.A greater value of permutation entropy, especially over **centro-posterior** regions, indexed a higher state of consciousness. |
| ([Piarulli et al., 2016](#_ENREF_21)) | MCS 6  VS/UWS 6 | Resting-state spectrum  Nonlinear analysis | Delta, theta and alpha power  Spectral entropy | 1.Higher delta power and lower theta, alpha, beta1 power were showed for the VS/UWS than the MCS group at **Pz**.  2.Spectral entropy and time variability of MCS was higher than that of the VS/UWS group at **Pz**. |
| ([Naro et al., 2018](#_ENREF_17)) | MCS 15  VS/UWS 17 | Resting-state spectrum  Functional connectivity | Delta, alpha and gamma power  dWPLI | 1.Patients with VS/UWS usually show lower gamma-power than patients with MCS.  2.Patients with MCS have some preserved topographical differentiation of alpha oscillations mainly in **parieto-occipital** regions, whereas patients with VS/UWS show residual multifocal alpha activities.  3.Gamma oscillatory activity in the parietal cortex correlates with the level of awareness.  4.Only the patients with MCS showed time-varying **centro-parietal** gamma dWPLI values. |
| ([Naro et al., 2016a](#_ENREF_18)) | HC 10  MCS 7  VS/UWS 6 | Resting-state spectrum | Delta, theta, alpha, beta and  gamma power | 1.**Parietal** theta, alpha, gamma source pattern was greater in the HC as compared to MCS and VS/UWS.  2.A positive correlation between the **parietal** theta, gamma source power and CRS-R scores.  3.More abnormal **parietal** source power of theta band in VS/UWS than MCS patients. |
| ([Babiloni et al., 2010](#_ENREF_1)) | HC 13  LIS 13 | Resting-state spectrum | Delta and alpha power | Compared to controls, patients with LIS had a lower power of alpha 2 (individual alpha frequency -2 to individual alpha frequency) and alpha 3 (individual alpha frequency to individual alpha frequency+2) and a higher delta power sources in **parietal** regions. |
| ([King et al., 2013](#_ENREF_11)) | HC 14  CS 24  MCS 68  VS/UWS 75 | Functional connectivity | Weighted symbolic mutual information | Weighted symbolic mutual information increases as a function of consciousness state and separates VS/UWS from MCS, particularly prominent across **centroposterior** areas. |
| ([Cai et al., 2020](#_ENREF_5)) | HC 23  MCS 19  VS/UWS 35 | Functional connectivity and network | Multiplex participation coefficient  Multiplex clustering coefficient | 1.Decreased multiplex participation coefficient values in MCS patients than VS/UWS, particularly in the **parietal-occipital** areas.  2.**Parietal-occipital** areas show remarkably increased multiplex clustering coefficient values in MCS and the controls compared to VS/UWS. |
| ([Cacciola et al., 2019](#_ENREF_4)) | MCS 13  VS/UWS 12 | Functional connectivity and network | Clustering coefficient | The beta band showed the most widespread alterations across many **parietal** region with a higher clustering coefficient in VS/UWS compared to MCS. |
| ([Chennu et al., 2017](#_ENREF_6)) | HC 26  LIS 4  EMCS 11  MCS+ 49  MCS− 17  VS/UWS 23 | Functional connectivity and network | Participation coefficients | High participation coefficients in **parietal** areas along with increasing level of consciousness. |
| ([Risetti et al., 2013](#_ENREF_23)) | MCS 3  VS/UWS 8 | Event related potential | nP3 (visual) | The nP3 in active condition is predominantly present in the posterior scalp regions (mainly **parietal**) in MCS, but not in VS/UWS. |
| ([Xiao et al., 2018](#_ENREF_35)) | HC 5  LIS 1  EMCS 1  MCS 5  VS/UWS 8 | Event related potential | P300 (visual) | The P300 component emerged in the central and parietal regions of HC, in the **centro-parietal** regions of the LIS but not emerged in DOC patients or emerged in other regions. |
| ([Wislowska et al., 2017](#_ENREF_34)) | HC 26  MCS 17  VS/UWS 18 | Resting-state spectrum | Theta and alpha power | In MCS patients, there were larger high-to-low frequency power ratio during day-time than during night-time over **parietal** midline electrode, while no significant in VS/UWS patients. |
| ([Rosanova et al., 2018](#_ENREF_26)) | HC 20  VS/UWS 16 | TMS-EEG | TMS evoked potentials | 1. TMS applied over **parietal** cortices, VS/UWS patients OFF-periods were ubiquitously observed and differed from awake healthy subjects stimulated over the same areas.  2. TMS applied over **parietal** cortices, the duration of the causal effects of TMS on local cortical activity in VS/UWS patients was shorter-lived than healthy awake controls. |
| ([Lee et al., 2022](#_ENREF_13)) | MCS 15  VS/UWS 15 | Resting-state EEG  TMS-EEG  Machine learning | Explainable consciousness indicator | The relevance score over the **parietal** region was higher than those in the frontal and temporal regions at the group level for both arousal and awareness. |
| ([Bai et al., 2021](#_ENREF_2)) | HC 25  MCS 25  VS/UWS 37 | Transient state | Fractional occupancy  Transition possibility | 1.DOC spent less time in the **posterior** state (activation in parietal lobe) than HC.  2.VS/UWS showed a decreased transition between the sensory and **posterior** states compared to MCS |
| ([Thibaut et al., 2021](#_ENREF_30)) | HC 33  VS/UWS 11  MCS* 15  MCS 54 | Resting-state spectrum  Functional connectivity and network | Theta，beta and alpha power  participation coefficient | MCS* patients had higher power in theta and alpha bands, lower power in delta band, higher alpha participation coefficient and alpha degree in **parietal** regions, compared to VS/UWS patients. |
| **Parietal** | **Subjects** | **EEG methodology** | **Main EEG characteristics** | **Main evidence** |
| **Evidence from DOC outcome** |  |  |  |  |
| ([Risetti et al., 2013](#_ENREF_23)) | MCS 3  VS/UWS 8 | Event related potential | nP3 (auditory) | The nP3 displayed a change in the topography, being largely distributed over the posterior (**parietal**) scalp regions in 2/4 VS/UWS who recovery to MCS (recovery time: half and 4 months). |
| ([Meiron et al., 2021](#_ENREF_16)) | HC 10  MCS 9  VS/UWS 1 | Event related potential | N1 (auditory) | The N1 of DOC survivors is stronger in the **central-parietal** cortex negativity than non-survivors. |
| ([Hermann et al., 2020](#_ENREF_9)) | EMCS 4  MCS 32  VS/UWS 24 | Resting-state spectrum  Non-linear analysis | Theta power  Permutation entropy | 1.Responsive patients showed a significant increase in normalized theta power with a topography maximal over the **parietal** cortices than non-responsive patients. after tDCS treatment.  2.A trend of an increase of permutation entropy (theta-alpha band) in responsive patients in the **parietal** region. |
| ([Chennu et al., 2017](#_ENREF_6)) | HC 26  LIS 4  EMCS 11  MCS+ 49  MCS− 17  VS/UWS 23 | Functional connectivity and network | Delta-connectivity  Participation coefficients  GOS-E | Strong connections across large parts of central and **parietal** areas were prominent in patients having negative outcomes as per the GOS-E(GOS-E≤2). |
| ([Cai et al., 2019](#_ENREF_6)) | MCS 18  VS/UWS 10 | Resting-state spectrum | alpha and delta power | Delta decreased and alpha increased at **P3, P4, Pz and POz** in the patients with consciousness recovery. |
| ([Zhang et al., 2022a](#_ENREF_38)) | MCS 29  VS/UWS 13 | Resting-state spectrum | alpha-beta power | Alpha-beta power increased, at the **parietal** electrodes, in the patients with consciousness recovery. |
| **Fronto-parietal connectivity** | **Subjects** | **EEG methodology** | **Evidence EEG characteristics** | **Main evidence** |
| **Evidence from DOC classification** |  |  |  |  |
| ([Naro et al., 2018](#_ENREF_9)) | MCS 15  VS/UWS 17 | Functional connectivity | dWPLI | Strength of connectivity within alpha **fronto-parietal** networks significantly correlates with the consciousness level. |
| ([Lehembre et al., 2012](#_ENREF_7)) | MCS 18  VS/UWS 10 | Functional connectivity | Coherence  Imaginary part of coherency  Phase lag index | Positive correlation between the connectivity of **frontal-posterior** (theta) and CRS-R. |
| ([Cavinato et al., 2015](#_ENREF_4)) | HC 15  MCS 14  VS/UWS 12 | Functional connectivity | Coherence | An increase (visual, auditory and noxious stimulation) of long-range **fronto-parietal** coherences at gamma band were in the controls and MCS, but not in VS/UWS. |
| ([Leon-Carrion et al., 2012](#_ENREF_8)) | MCS 7  SND 9 | Functional connectivity | Coherence  Granger causality | The SND had a higher number of functional connections between **frontal and parietal/occipital** regions than MCS. |
| ([Cacciola et al., 2019](#_ENREF_3)) | MCS 13  VS/UWS 12 | Functional connectivity | Peak lagged phase synchronization | VS/UWS had lower functional connectivity than MCS patients, mainly involving the inter-hemispheric **fronto-parietal** connectivity patterns. |
| ([Chennu et al., 2017](#_ENREF_5)) | HC 26  LIS 4  EMCS 11  MCS 66  VS/UWS 23 | Functional connectivity and network | Participation coefficients | Stronger inter-hemispheric connectivity of alpha between **frontal** and **parietal** areas, along with increasing level of consciousness  MCS- and MCS + patients showed similar levels of connectivity, but the topographical pattern in MCS + patients showed the presence of a discernible **frontoparietal** focus for the strongest connections. |
| ([Naro et al., 2020](#_ENREF_10)) | MCS 15  VS/UWS 17 | Functional connectivity and network | Multilayer and multiplex network analysis | The deterioration and heterogeneity of the **frontal-parietal** network were the discriminant between patients with MCS and VS/UWS, with UWS patients being more severe. |
| ([Wang et al., 2022](#_ENREF_12)) | HC 30  MCS 76  VS/UWS 105 | TMS-EEG | TMS evoked potentials | MCS shows significant TMS-evoked excitability from the **frontal region to parietal** and occipital regions lasting to about 200ms, while the VS/UWS patient has no responses to TMS. |
| ([Bai et al., 2018](#_ENREF_2)) | MCS 8  VS/UWS 9 | Functional connectivity | Coherence | **Fronto-parietal** coherence significantly increased in the theta band and decreased in the gamma band in the MCS group, while no significant changes in the VS/UWS group after tDCS. |
| ([Bai et al., 2021](#_ENREF_1)) | HC 25  MCS 25  VS/UWS 37 | Transient state | State dependent coherence | Coherence in the alpha band between the **medial prefrontal cortex and posterior cingulate cortex** was found to be disrupted in DOC compared to healthy subjects in the anterior state. |
| **Fronto-parietal connectivity** | **Subjects** | **EEG methodology** | **Evidence EEG characteristics** | **Main evidence** |
| **Evidence from DOC outcomes** |  |  |  |  |
| ([Schorr et al., 2016](#_ENREF_11)) | HC 24  MCS 15  VS/UWS 58 | Functional connectivity | Coherence | Parietal coherence was significantly higher in delta, theta, alpha and beta frequencies between **frontal and parietal** regions in the improved group (VS/UWS to MCS) after one year later. |

Note: HC = healthy control; LIS = locking in syndrome; SND = severe neurocognitive disorders; CS = conscious state; EMCS = emerged from minimally conscious state; MCS = minimally conscious state; VS/UWS = vegetative consciousness/unresponsive wakefulness syndrome; dWPLI = debiased-weighted phase lag index; QPSC = Quadratic phase self-coupling; CRS-R = revised version of the coma recovery scale; GOS-E = Glasgow Outcome Scale-Extended;
